# The C-terminal coiled-coil domain of *Corynebacterium diphtheriae* DIP0733 is crucial for interaction with epithelial cells and pathogenicity in invertebrate animal model systems

**DOI:** 10.1186/s12866-018-1247-z

**Published:** 2018-09-04

**Authors:** Dulanthi Weerasekera, Franziska Stengel, Heinrich Sticht, Ana Luíza de Mattos Guaraldi, Andreas Burkovski, Camila Azevedo Antunes

**Affiliations:** 1 Microbiology Division, Friedrich-Alexander-University Erlangen-Nuremberg, Erlangen, Germany; 2 Division of Bioinformatics, Institute of Biochemistry, Friedrich-Alexander-University Erlangen-Nuremberg, Erlangen, Germany; 3 Laboratory of Diphtheria and Corynebacteria of Clinical Relevance-LDCIC, Faculty of Medical Sciences, Rio de Janeiro State University, Rio de Janeiro, Brazil

**Keywords:** *Caenorhabditis elegans*, Corynebacteria, *Galleria mellonella*, Host pathogen interaction

## Abstract

**Background:**

*Corynebacterium diphtheriae* is the etiologic agent of diphtheria and different systemic infections. The bacterium has been classically described as an extracellular pathogen. However, a number of studies revealed its ability to invade epithelial cells, indicating a more complex pathogen-host interaction. The molecular mechanisms controlling and facilitating internalization of *C. diphtheriae* still remains unclear. Recently, the DIP0733 transmembrane protein was found to play an important role in the interaction with matrix proteins and cell surfaces, nematode colonization, cellular internalization and induction of cell death.

**Results:**

In this study, we identified a number of short linear motifs and structural elements of DIP0733 with putative importance in virulence, using bioinformatic approaches. A C-terminal coiled-coil region of the protein was considered particularly important, since it was found only in DIP0733 homologs in pathogenic *Corynebacterium* species but not in non-pathogenic corynebacteria. Infections of epithelial cells and transepithelial resistance assays revealed that bacteria expressing the truncated form of *C. diphtheriae* DIP0733 and *C. glutamicum* DIP0733 homolog are less virulent, while the fusion of the coiled-coil sequence to the DIP0733 homolog from *C. glutamicum* resulted in increased pathogenicity. These results were supported by nematode killing assays and experiments using wax moth larvae as invertebrate model systems.

**Conclusions:**

Our data indicate that the coil-coiled domain of DIP0733 is crucial for interaction with epithelial cells and pathogenicity in invertebrate animal model systems.

**Electronic supplementary material:**

The online version of this article (10.1186/s12866-018-1247-z) contains supplementary material, which is available to authorized users.

## Background

*Corynebacterium diphtheriae* is the causative agent of diphtheria, a toxemic infection of the upper respiratory tract [1]. Notably, *C. diphtheriae* is present on the list of the most important global pathogens. These pathogens cause global fatalities, and are often part of mixed infections or cause multiple different diseases, making them difficult to identify [2]. Besides classical diphtheria, an increasing number of systemic infections caused by non-toxigenic *C. diphtheriae* strains have been reported [3–7]. Moreover, it has been demonstrated that *C. diphtheriae* possesses various virulence factors that may act independently of diphtheria toxin [8–12]. Among these, only a few have been investigated in detail.

The DIP0733 protein - initially described as a non-fimbrial surface protein and designated as 67-72p [13] - was recently characterized as a multi-functional virulence factor of *C. diphtheriae* [14]. This protein plays an important role in the interaction process with extracellular matrix proteins and cell surfaces, nematode colonization, cellular internalization and induction of cell death [13, 14]. Despite its multiple roles in pathogenicity, DIP0733 was found to lack annotated functional domains within the protein besides seven transmembrane helices [14]. Therefore, the aim of this work was to identify functional elements in DIP0733 and its homologs and investigate how they contribute to the process of host pathogen interaction. In this study, short linear motifs and structural elements of DIP0733 with putative importance in virulence were identified using bioinformatic approaches. A C-terminal coiled-coil region of the protein was considered particularly important, since it was found only in DIP0733 homologs of pathogenic but not in non-pathogenic corynebacteria. Mutagenesis studies including the construction of truncated and variant forms of DIP0733 and their expression by its *C. diphtheriae* mutant strain were carried out and their involvement in interactions with biotic and abiotic surfaces were investigated. Furthermore, their interaction with epithelial cells and their influence in transepithelial resistance as well as with *Caenorhabditis elegans* and *Galleria mellonella,* invertebrate animal model systems [15], were also studied.

## Methods

### Bacterial strains and culture conditions

*Escherichia coli* OP50, *E. coli* DH5αMCR and *Salmonella enterica* serovar Typhimurium NCTC 12023 were grown in Luria Bertani (LB) medium at 37 °C [16]. *C. diphtheriae* strains were grown in heart infusion (HI) at 37 °C with constant shaking at 125 r.p.m. When appropriate, 50 μg kanamycin ml^− 1^ or 25 μg chloramphenicol ml^− 1^ were added to the medium. Bacterial strains, cell lines and plasmids used in this study are listed in Table 1.

**Table 1:** Strains, cell lines, plasmids and primers used in this study

Strain	Description	Source
*Escherichia coli*		
DH5αMCR	*endA1 supE44 thi-1* λ- *recA1 gyrA96 relA1 deoR ∆(lacZYA-argF) U196 Ø*80 ∆*lacZ∆M15mcrA* ∆ *(mmrhsdRMS mcrBC)*	[39]
OP50	Uracil-auxotrophic *E. coli* B strain	[40]
*Salmonella enterica* serovar Typhimurium		
NCTC 12023	Wild-type identical to ATCC 14028	National Collection of Type Cultures (Colindale, UK)
*Corynebacterium diphtheriae*		
CDC-E8392	Wild-type, Biovar. *mitis*, tox^+^	[41]
CAM-1	CDC-E8392 *DIP0733::*pCR2.1 TOPO*DIP0733*’	[14]
INCA 402	Isolate from a patient with pneumonia, biovar. *Belfanti*, tox^−^	[41]
*Corynebacterium glutamicum*		
ATCC 13032	Type strain, non-pathogenic	[42]
Cell lines		
Detroit562	Human hypopharyngeal carcinoma cells (ATCC CCL-2)	[43]
HeLa	Human cervical carcinoma cells (ATCC CCL-2)	[44, 45]
Plasmids		
pXMJ19	*ori colE1*, *oriCg*, *ptac*, Cm^R^	[46]
pXMJ19_DIP0733	*ori colE1*, *oriCg*, *ptac*, *CDCE8392_0678*, Cm^R^	[14]
pXMJ19_DIP0733-cc	pXMJ19 with 2700 bp insert of 3′-shortened *CDCE8392_0678* (DIP0733 homolog without sequence for *C*-terminal *disorder*- and *coiled-coil*-region) from *C. diphtheriae* CDC-E8392	This study
pXMJ19_Cg0896	*ori colE1*, *oriCg*, *ptac*, *Cg0896*, Cm^R^	
pXMJ19_Cg0896 + cc	pXMJ19 with 2693 bp insert of 3′-shortened *Cg0896* (DIP0733 homolog without sequence for *C*-terminal *disorder*- and *coiled-coil*-region) from *C. glutamicum* ATCC 130323 and 268 bp insert of 5`-shortened *CDB402_0643* (DIP0733 homolog sequence for *C*-terminal *disorder*- and *coiled-coil*-region) from *C. diphtheriae* INCA-402	
Primers (5`- 3`)		
Probe 16SrRNA	GCAGCCGCGGTAATACGTAG	This study
	GGGCCCTAATACGACTCACTATAGGGACATCTCACGACACGAGCTG	
Probe DIP0733	CGACCCAGTGCTTAAGGCAT	
	GGGCCCTAATACGACTCACTATACCTGTGCCTGCTTTGGACCC	
Probe Cg0896	GTACGACGGAACTGTTGAAC	
	GGGCCCTAATACGACTCACTATAGCCTGCTTTGGACCCTGGGTC	
pXMJ19_DIP0733-cc	CGCG*CCTGCA*GGTTGGCGACCGGTTTTACGCG	
	CGCG*CCCGGG*TTACTTAGGGTCAATACCTACCT	
pXMJ19_Cg0896	CGCG*CCTGCA*GGTTGTCGACTGGTCTCACACC	
	CGCG*CCCGGG*CTACTGTGCGGACTGGTAGC	
pXMJ19_Cg0896 + cc	CGCGCCTGCAGGTTGTCGACTGGTCTCACACC	
	CGCG*CCTTA*GGCTGCGATAGGGCTTCAG	

### Bioinformatic analysis of short linear motifs

In order to find functional structural units of the DIP0733, deposited information about the nucleotide and amino acid sequences were collected from databases. The DIP0733 protein sequences of 5 corynebacteria (*C. diphtheriae* CDC-E8392 and INCA-402, *Corynebacterium ulcerans* BR-AD22 and *Corynebacterium pseudotuberculosis* 258 as pathogenic strains and *Corynebacterium glutamicum* ATCC 13032 as a non-pathogenic species) were aligned using the compositional substitution matrix adjustment method executed on BLASTp (Additional file 1: Figure S1). Subsequently, the DIP0733 homolog sequences were examined by means of the Eukaryotic Linear Motif (ELM) database for short linear motifs (SLiMs). These motifs are categorised by different functions, such as CLV (cleavage sites), DEG (degradation sites), DOC (docking sites), LIG (ligand binding sites), MOD (post-translational modification sites) and TRG (targeting sites). The large number of possible linear motifs could be reduced by considering only highly conserved motifs. In addition, all motifs were compared, and those which were present only in pathogenic strains and not in non-pathogenic corynebacteria were considered in more detail. Subsequently, the presence and position of a coiled-coil area in different strains predicted with ELM database was considered for the construction of the recombinant DIP0733 mutant variants.

### Molecular biology techniques and construction of recombinant DIP0733 variants

**Fig. 1:**
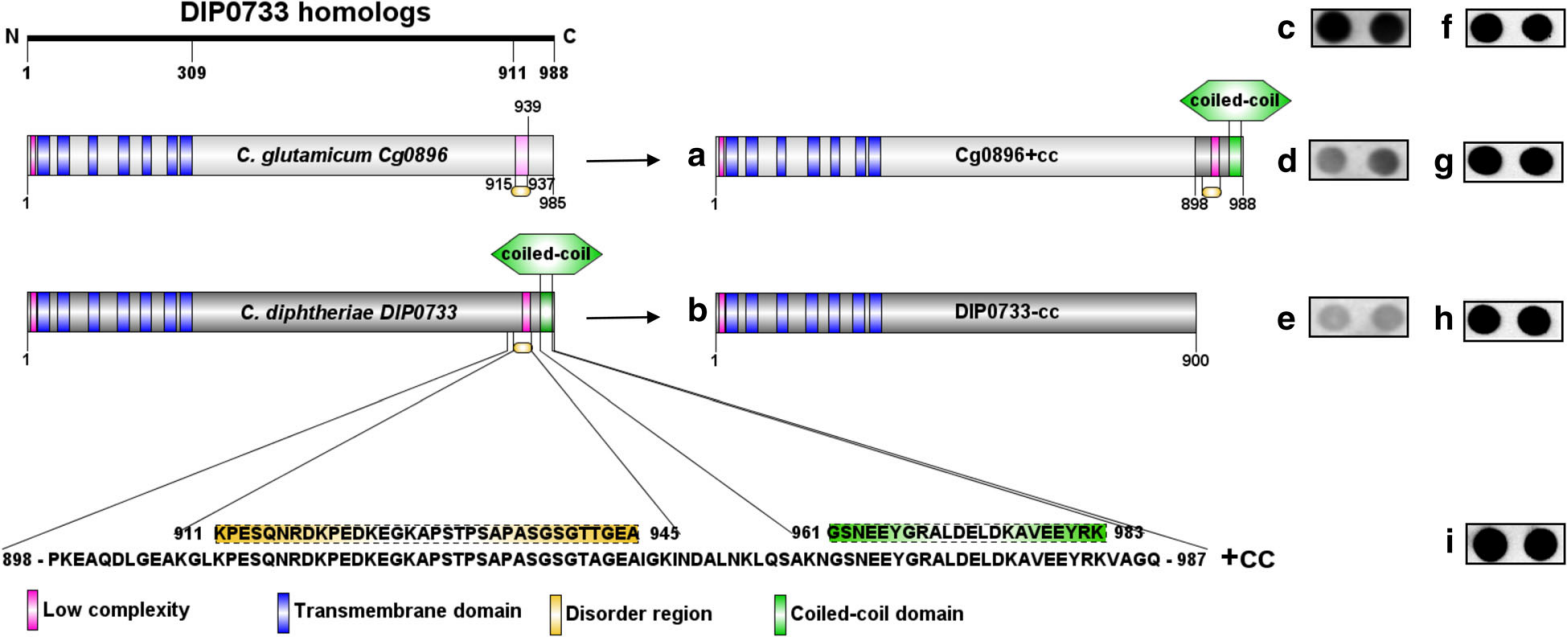
Schematic representation of DIP0733 homologs and their truncated forms. Left: DIP0733 homologs in non-pathogenic (*C. glutamicum*) and pathogenic (*C. diphtheriae*) corynebacteria and their common features: low complexity regions (pink boxes), transmembrane helix domains (blue boxes), disorder regions (yellow boxes) and coiled-coil domain (green boxes). Right: strategy for the cloning of the truncated DIP0733 variants into pXMJ19. The *C. glutamicum* DIP0733 homolog sequence with the fused coding sequence for the coiled-coil domain from *C. diphtheriae* is represented by Cg0896 + cc (**a**). DIP0733 homolog sequence from *C. diphtheriae* without the coiled-coil domain and disorder region are represented by DIP0733-cc (**b**). Similar transcription levels of the DIP0733 homologs (Cg0896, Cg0896 + cc, DIP0733-cc) are demonstrated by RNA hybridization with the respective probes for DIP0733 homologs (**c**-**e**) and 16SrRNA respectively (**f**-**h**). The transcription levels of the control strains transformed with the empty plasmid was detected for 16SrRNA probe (**i**) and no signal for DIP0733 homolog probes was observed (data not shown)

Standard molecular biology techniques were used for plasmid isolation, transformation of *E. coli* and cloning [16]. *C. diphtheriae* CDC-E8392 wild-type, the CAM-1 DIP0733 mutant strain [14] and their respective complementation and overexpression strains were used in this study. Additionally, different complementation plasmids encoding three recombinant forms of DIP0733 homologs were constructed (Table 1). The first comprised the complete nucleotide sequence of DIP0733 homolog from *C. glutamicum* ATCC 13032 and was designated as pXMJ19_Cg0896, according to its gene identifier. The second plasmid encoded a DIP0733 hybrid variant with the sequence of the *C. diphtheriae* C-terminal coiled-coil region fused downstream to *Cg0896* and it was consequently designated as pXMJ19_Cg0896 + cc*.* The third plasmid was constructed to encode a C-terminal truncated *C. diphtheriae* DIP0733 homolog lacking its coiled-coil region and was designated as pXMJ19_DIP0733-cc (Fig. 1a-e and Table 1). For easier comprehension, the constructs harbouring the sequences of DIP0733 homologs from *C. diphtheriae* remained named as DIP0733. Hence, the DIP0733 homolog sequences from the *C. diphtheriae* strains used in this study share high similarity with DIP0733 from NCTC 13129 (Additional file 1: Figure S1).

The DIP0733 homolog nucleotide sequences from *C. diphtheriae* and *C. gutamicum* were amplified via PCR with primers containing specific restriction sites as described in Table 1, and the products were digested for 1 h at 37 °C. The vector pXMJ19 was digested with the same restriction enzymes as the respective inserts and dephosphorylated for 30 min at 37 °C. Ligations of inserts and pXMJ19 DNA were carried out with T4 DNA ligase overnight at 22 °C. After transformation of *E. coli* DH5αMCR, positive clones were selected on LB medium containing 25 μg chloramphenicol ml^− 1^.

The resulting plasmids (Table 1) were isolated, sequenced and 1 μg of plasmid DNA was used to transform electrocompetent *C. diphtheriae* CAM-1 strain [14]. Transformants were selected on HI agar containing 12.5 μg chloramphenicol ml^− 1^ and 25 μg kanamycin ml^− 1^. For verification of the transcription levels of the above mentioned DIP0733 constructions, RNA isolation and hybridization were carried out as previously described [17]. For hybridization of the digoxigenin-labelled RNA probes (oligonucleotide primers for the different probes are listed in Table 1) and its detection, alkaline phosphatase-conjugated anti-digoxigenin Fab fragments and CSPD (Disodium 3-(4-methoxyspiro {1,2-dioxetane-3,2′-(5′-chloro)tricyclo [3.3.1.1^3,7^]decan}-4-yl)phenyl phosphate) as the light-emitting substrate were used as suggested by the supplier (Roche, Mannheim, Germany). Chemiluminescence was detected with a ChemiDoc XRS+ system (BioRad, Munich, Germany).

### Extracellular matrix (ECM) and plasma protein binding assays

ECM protein binding assays were performed based on the combination of two protocols [7, 18] as previously described [12]. In summary, 96-well polystyrene microtiter plates were coated with collagen type I, collagen type IV and fibrinogen (Sigma, Munich, Germany) dissolved in PBS (Phosphate Buffered Saline) and incubated at 4 °C for 24 h. The coated wells were washed twice with PBS and subsequently filled with 200 μl of resuspended bacterial cells in HI medium (OD_600_ of 0.2). All experiments were carried out in triplicate. The biofilm formation occurred after incubation for 24 h at 37 °C under static conditions. Unattached bacterial cells were discarded and the wells were washed three times with PBS. Biofilms were heat-fixed for 1 h, stained with crystal violet for 15 min at room temperature and rinsed with distilled water. Plates were air dried for 1 h at 37 °C followed by re-solubilization of the stained biofilms in glacial acetic acid. The biofilm was quantified by spectrophotometry with the plate reader TECAN infinity F200 PRO (TECAN, Crailsheim, Germany), using an optical density of 620 nm.

### Cell culture experiments

Detroit562 cells were cultivated in Dulbecco’s modified Eagle’s medium (DMEM), high glucose with L-glutamine and sodium pyruvate (PAA Laboratories, Austria), supplemented with 120 μg penicillin ml^− 1^, 120 μg streptomycin ml^− 1^ and 10% (*v*/v) heat-inactivated fetal bovine serum (FBS) (Life Technologies, Germany) in a CO_2_ incubator. HeLa cells were cultured in DMEM, high glucose with L-glutamine (PAA Laboratories, Austria) supplemented with 100 μg gentamicin ml^− 1^ and 12 μg ciprofloxacin ml^− 1^ and 10% heat-inactivated FBS in a CO_2_ incubator. Cells were passaged at a ratio of 1:10 twice per week.

### Adhesion and invasion assays

Cellular interaction assays were performed using epithelial cells derived from human cervical carcinoma as previously described protocols [12, 14]. In short, HeLa cells were infected with *C. diphtheriae* strains, washed with PBS, detached with trypsin solution, lysed with Tween 20 and the number of colony forming units (CFU) for adhesion was determined. Invasion of epithelial cells was determined as for the above mentioned adhesion assay, followed by further treatment with gentamicin for 90 min. The relative efficiency of invasion (%) was calculated based on the ratio of CFU prior to infection and CFU on the lysate plates after infection, multiplied by 100.

### Measurement of transepithelial resistance

Transepithelial resistance assays were carried out in a modified version of a formerly described protocol [17]. Briefly, 1 × 10^5^ Detroit562 cells were seeded per transwell and cultivated in DMEM for 2 weeks. Bacteria were grown until the beginning of the exponential phase was reached. 100 μl of resuspended bacterial cells in PBS (OD_600_ 5) were used to inoculate the transwells. All experiments were carried out in triplicate. After infection, the transepithelial resistance of Detroit562 cells was measured using a volt-ohm-meter (EVOM2, World Precision Instruments, Berlin, Germany). The putative detrimental effect of excessive bacterial growth towards Detroit562 cells was avoided by the replacement of the supernatant of infected cells with fresh DMEM for overnight incubation.

### *C. elegans* survival assay

The assays were performed as previously described [12, 19–23]. Briefly, *C. elegans* N2 were maintained and synchronized on plates containing nematode growth medium (NGM) agar for approximately four to seven days at 20 °C and used in infection assays with *C. diphtheriae* strains. Twenty L4 stage larval worms were infected with 20 μl of each bacterial strain (OD_600_ 1.0) on NGM plates at 20 °C for 24 h. Worms were assessed each day following infection and the dead nematodes were counted and removed every 24 h. For each strain, approximately 60 nematodes were used in the experiments. *E. coli* OP50 was used as a control. *C. elegans* N2 was maintained on *E. coli* OP50 for six to seven days until the worms became starved, as indicated by clumping behaviour [20]. For the Kaplan-Meier survival plots, the Mantel-Cox log-rank test (95% CI) was used for comparing the survival distributions between the DIP0733 mutant strain CAM-1 or the wild-type strain CDC-E8392 versus each mutant variant construct, obtaining the average survival times and *p-*values of less than 0.05 considered significant. The data and all statistical analysis was performed with Prism 7.0 (GraphPad, CA, USA).

### Infection and monitoring of *G. mellonella*

Infections of wax moth (*G. mellonella*) larvae were carried out as described previously [15]. In short, bacteria from an overnight culture were inoculated in fresh medium and grown until an OD_600_ of 0.6 was reached. Bacteria were harvested by centrifugation (10 min, 4500 x g) and resuspended in 10 mM MgSO_4_ to an OD_600_ of 10 (approximately 3 × 10^9^ CFU ml^− 1^). For the infection, a 50 μl Hamilton syringe was used to inject 5 μl aliquots into *G. mellonella* larvae via the hindmost left proleg. Larvae were incubated at 25 °C and pictures were taken on the third day of infection. Post-infection, *G. mellonella* larvae were monitored daily during 5 days for their activity, melanization and survival. A score was provided for each monitored attribute that supported a global health index of each individual wax worm (Additional file 2: Table S1; adapted from the Health Index Scoring System Table from [24]). Healthy, uninfected wax moth larvae typically score between 10 and 11, while infected dead larvae score between 0 and 1.5. For the health index scores of the wax moth larvae infected with *C. diphtheriae* strains, the 2-way ANOVA column statistics were calculated comparing values between the DIP0733 mutant strain CAM-1 or the wild-type strain CDC-E8392 versus each mutant variant construct. *P-*values of less than 0.05 considered significant.

## Results

### Analysis of short linear motifs of DIP0733 homologs

DIP0733 proteins are composed of seven transmembrane helices and a hydrophilic region. In order to unravel more structural and functional information, the protein sequence of DIP0733 homologs were examined by means of the ELM database for short linear motifs (SLiMs), which can be involved in different functions categorized from a repository of experimentally validated linear motif classes and instances that were manually curated from the literature [25]. SLiMs are functional segments of the protein sequence that are of fundamental importance for numerous biological processes by mediating protein–protein interactions, regulating the dynamic processes involved in cell signaling [26]. Almost seventy SLiMs were found along DIP0733 and its homologs. Among them, important linear motifs comprising docking sites, ligand binding sites and post-translational modification sites, such as DOC_MAPK_JIP1_4 (MAPK docking motifs or kinase interaction motif (KIM)), LIG_eIF4E_1 (motif binding to eIF4E, a key regulator of eukaryotic cap-dependent translation), LIG_SH3_4 (recognized by those SH3 domains with a non-canonical class II recognition specificity), LIG_SUMO_SIM_anti_2 (SUMO-interacting motif (SIM)), LIG_TYR_ITSM (ITSM (immunoreceptor tyrosine-based switch motif), MOD_PK_1/MOD_PKA_1 (phosphorylase kinase phosphorylation site) and MOD_SUMO_for_1 (sumoylation site), were exclusively found in DIP0733 homologs from pathogenic corynebacteria. Furthermore, the C-terminal domain of DIP0733 is characterized by a pronounced coiled-coil region that is likely to be located extracellularly (Fig. 1). This coiled-coil domain was found to be present in all *C. diphtheriae* and *C. pseudotuberculosis* strains between amino acid positions 961 and 983 and 951 and 978, respectively, whereas it was not found in *C. glutamicum* and *C. ulcerans* DIP0733 homologs. This led to the idea that the virulence properties of DIP0733 in *C. diphtheriae* could be influenced by its C-terminal coiled-coil region and consequently, we constructed truncated variants (Fig. 1) to study the function of this domain.

### Binding properties to collagen and fibrinogen

In previous studies, the interaction of *C. diphtheriae* with proteins of the extracellular matrix surrounding eukaryotic cells, e. g. collagen I and IV, was reported [12, 14, 19, 27]. Additionally, the interaction with fibrinogen, a major component of the human plasma, which is crucial for blood clot formation due to its conversion into insoluble fibrin, has also been investigated in corynebacteria [27–29].

**Fig. 2:**
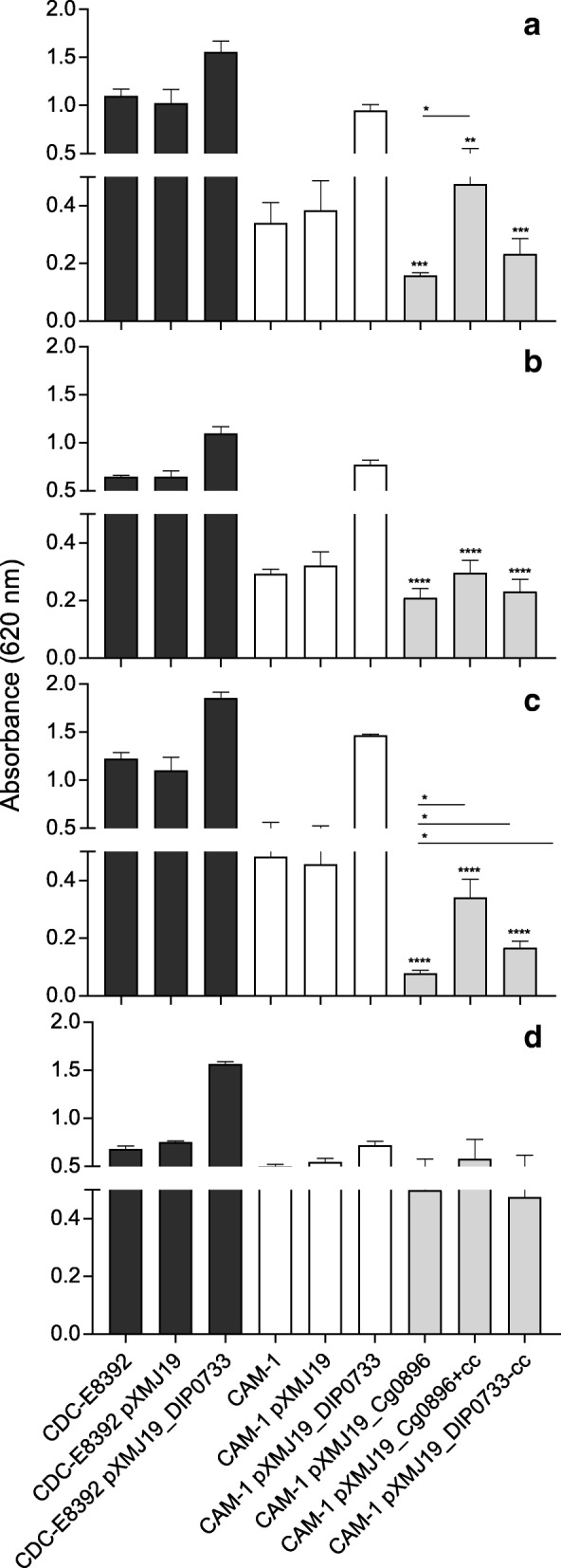
Binding to extracellular matrix proteins and fibrinogen. Binding to human type I collagen (**a**) and type IV collagen (**b**), fibrinogen (**c**) and polystyrene (**d**) by *C. diphtheriae* strains. Dark grey and white columns represent reproduced assays with strains CDC-E8392 and CAM-1 respectively as previously shown [14]. Grey columns represent assays performed with new constructs for different truncated DIP0733 homologs (see Fig. 2). Data shown represent mean values ± SD of at least three independent biological replicates. Statistically relevant differences from CAM-1 pXMJ19_DIP0733 and its respective truncated forms (light grey columns) were based on Unpaired Student’s t-test values below 0.05, 0.01, 0.001 and 0.0001 are indicated by one, two, three and four asterisks, respectively. Unpaired Student’s t-test was performed using GraphPad Prism 7.0 (GraphPad, CA, USA)

To test the bacterial binding to extracellular matrix components and plasma proteins, the strains were incubated in microtitre plate wells previously coated with the corresponding proteins. Compared to the wild-type strain CDC-E8392, the binding of the DIP0733 mutant strain CAM-1 to collagen was significantly impaired (Fig. 2a, b). The binding level of CDC-E8392 to collagen type I was significantly higher (A_620_ 1.098 ± 0.128) than CAM-1 (A_620_ 0.34 ± 0.123). In the case of the overexpression strain, CDC-E8392 pXMJ19_DIP0733 and complementation strain CAM-1 pXMJ19_DIP0733, binding of collagen type I resulted in enhanced rates (A_620_ 1.554 ± 0.205 versus A_620_ 0.946 ± 0.107, respectively). These results supported the findings of the previous study [14]. The strains CAM-1 pXMJ19_Cg0896 and CAM-1 pXMJ19 DIP0733-cc showed a reduction in binding to collagen type I (A_620_ 0.158 ± 0.018 and A_620_ 0.233 ± 0.091, respectively). In contrast, CAM-1 pXMJ19_Cg0896 + cc showed an increased binding to collagen type I (A_620_ 0.475 ± 0.136) (Fig. 2a).

The pattern of binding to collagen type I was similar to the collagen type IV, when considering the complementation and overexpression behavior. CDC-E8392 pXMJ19_DIP0733 showed a higher binding rate of A_620_ 1.095 ± 0.122 compared to the wild-type CDC-E8392 (A_620_ 0.647 ± 0.023) and the binding of the full complementation strain to type IV collagen (A_620_ 0.7709 ± 0.046) presented similar value compared to CDC-E8392 and a significant higher binding than the mutant strain CAM-1 (A_620_ 0.293 ± 0.028 for CAM-1). The mutant variants showed a reduction in binding to collagen type IV; however, a significant difference in binding among the three strains were not observed (Fig. 2b).

The binding to fibrinogen was also reduced by the mutant compared to the wild-type (A_620_ 1.223 ± 0.113 for CDC-E8392 versus A_620_ 0.483 ± 0.134 for CAM-1; Fig. 2c). Similar to collagen type I, the binding to fibrinogen by the overexpression and complementation strains were higher compared to CDC-E8392 and CAM-1 (A_620_ 1.855 ± 0.102 for CDC-E8392 pXMJ19_DIP0733 versus A_620_ 1.466 ± 0.024 for CAM-1 pXMJ19_DIP0733). In contrast, the binding to fibrinogen was reduced by the strain expressing DIP0733 with a truncated coiled-coil domain (A_620_ 0.167 ± 0.04 for CAM-1 pXMJ19_DIP0733-cc). The strain CAM-1 pXMJ19_Cg0896 + cc showed about 4-fold higher binding to fibrinogen compared to CAM-1 pXMJ19_Cg0896 (A_620_ 0.340 ± 0.111 versus A_620_ 0.079 ± 0.016).

The binding to polystyrene surfaces by CDC-E8392 (A_620_ 0.680 ± 0.032) and its DIP0733 overexpression strain (A_620_ 1.563 ± 0.024) indicated that the whole DIP0733 protein could be involved in binding to abiotic surfaces. Moreover, the mutant strain CAM-1 presented a reduced ability to bind to polystyrene compared to the wild-type CDC-E8392 and its complementation strain (A_620_ 0.502 ± 0.020 for CAM-1 and A_620_ 0.680 ± 0.032 for CAM-1 pXMJ19_DIP0733). However, no significant difference in the binding to polystyrene surfaces was observed among the mutant variant strains, suggesting that the coiled-coil region is not interfering significantly with this process (Fig. 2d).

### Influence on adhesion of *C. diphtheriae* to epithelial cells

**Fig. 3:**
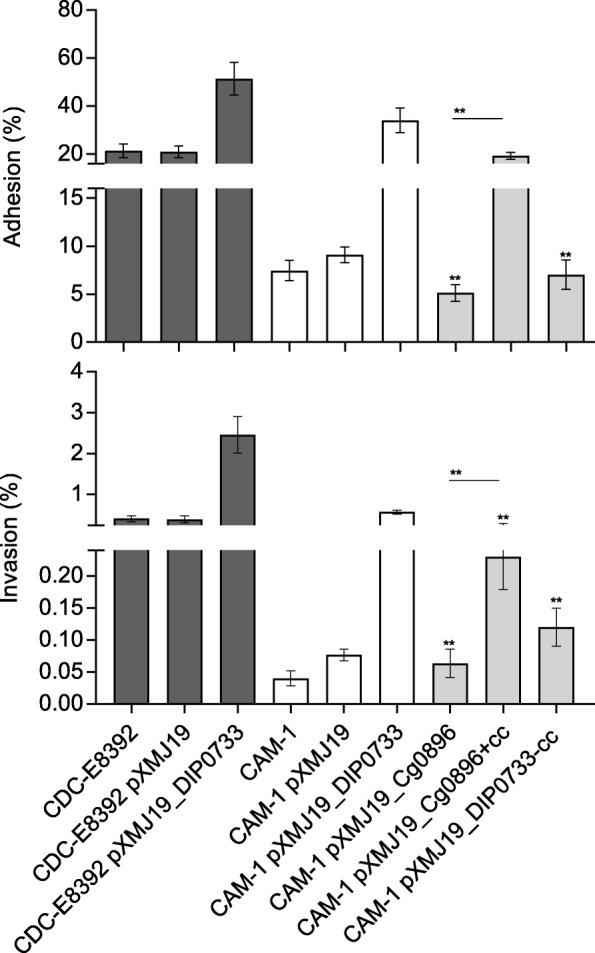
Adhesion to and invasion of epithelial cells. Dark grey and white columns represent reproduced assays with strains previously showed by [14]. Grey columns represent the assays performed with new constructs for different truncated DIP0733 homologs (see Fig. 1). Statistically relevant differences between CAM-1 pXMJ19_DIP0733 and its respective truncated forms (light grey columns) were based on Unpaired Student’s t-test. Values below 0.05, 0.01, 0.001 and 0.0001 are indicated by one, two, three and four asterisks, respectively. Adhesion and invasion are expressed as percentage of the inoculum, showing means and SD of at least three independent biological replicates

The function of DIP0733 in host cell contact was quantitatively analyzed using wild-type and the mutant strain CAM-1 expressing recombinant versions of the protein in HeLa epithelial cell adhesion assays (Fig. 3a). While CDC-E8392 reached an adhesion rate of 21.4 ± 5%, the CAM-1 attachment was reduced to only 7.5 ± 1.8%. The rates were unchanged when the corresponding strains were transformed with plasmid pXMJ19 for control (20.9 ± 4.1% for the wild-type and 9.1 ± 1.4% for CAM-1). The overexpression of DIP0733 led to an adhesion rate of 51.5 ± 11.8% for the wild-type and 34.1 ± 9% for the mutant CAM-1. Interestingly, the strain CAM-1 pXMJ19_Cg0896 + cc, expressing the DIP0733 homolog of *C. glutamicum* fused with the C-terminal coiled-coil region from *C. diphtheriae*, showed an enhanced adherence rate of 19.2 ± 2.4% compared to the strain CAM-1 pXMJ19_Cg0896 (5.1 ± 1.5%).

### Influence on invasion towards epithelial cells

When the internalization of the wild-type and the DIP0733 mutant strains were determined using gentamicin protection assays, the mutant showed a significant decreased internalization by HeLa cells. An invasion rate of 0.4 ± 0.12% was reached by the wild-type CDC-E8392 and 0.04 ± 0.02% by the DIP0733 mutant strain CAM-1 (Fig. 3b). As in the case of adhesion assays, transformation of the wild-type or CAM-1 with the vector pXMJ19 (empty vector control) had no influence on invasion rates (0.39 ± 0.15% and 0.08 ± 0.02% respectively). In contrast, transformation with the DIP0733 expression plasmid pXMJ19-DIP0733 resulted in increased invasion rates, which exceeded the wild-type rates by a factor of about four for the wild-type and about three for the mutant. As in the case of adherence, the internalization rate of the CAM-1 pXMJ19_DIP0733-cc strain was reduced (0.12 ± 0.05%). Moreover, the CAM-1 pXMJ19_Cg0896 + cc strain showed an invasion rate of about 0.16 ± 0.04% whereas the CAM-1 pXMJ19_Cg0896 strain had an invasion rate of only 0.01 ± 0.01%. The results obtained suggested a direct function of the DIP0733 protein in respect to the internalization of *C. diphtheriae* by epithelial cells.

### Transepithelial resistance

**Fig. 4:**
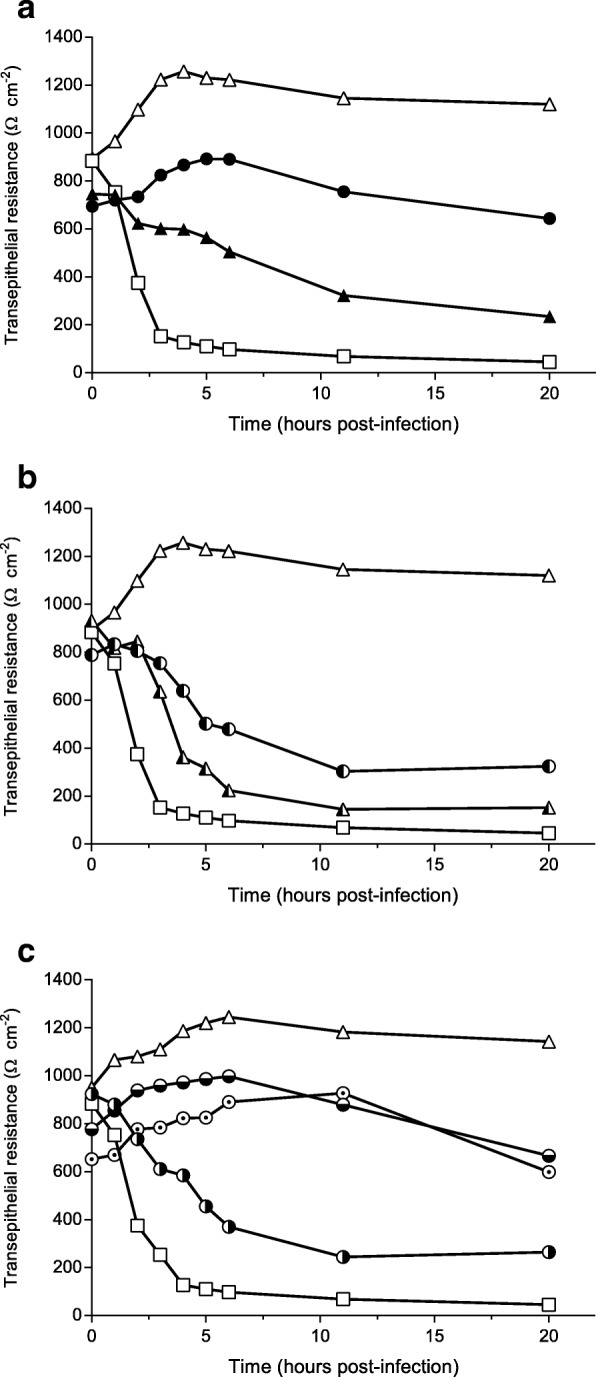
Transepithelial resistance measurements. Polarized Detroit562 monolayers were grown on transwells and infected with *C. diphtheriae* strains CDC-E8392 (▲) and CAM-1 (●) (**a**); CDC-E8392 pXMJ19_DIP0733 (◭) and CAM-1 pXMJ19_DIP0733 (◐) (**b**); CAM-1 pXMJ19_Cg0896 (⊙), CAM-1 pXMJ19_DIP0733-cc (◒) and CAM-1 pXMJ19_Cg0896 + cc (◑) (**c**). For control, cells were incubated without (Δ) bacteria and (□) *S.* Typhimurium (**a**-**c**). Experiments were carried out independently in triplicate and typical results are shown

Some pathogens can cause severe damage on cell membranes and the transepithelial resistance of cell monolayers is dramatically reduced due to the loss of cell integrity [17, 30]. In this study, *S*. Typhimurium NCTC 12023 was used as a positive control to test the influence of *C. diphtheriae* strains on transepithelial resistance (Fig. 4). The negative control without bacteria showed a gradual increase in the transepithelial resistance for about 4 h and later stayed constant up to 20 h, whereas the infection of Detroit562 monolayers with *S*. Typhimurium caused a fast breakdown of transepithelial resistance within 2 h. Compared to *S*. Typhimurium, the detrimental effects caused by *C. diphtheriae* were considerably lower. However, the cells infected with the wild-type CDC-E8392 showed a faster breakdown of transepithelial resistance within 6 h in comparison to the mutant CAM-1. Conversely, the cells infected with the mutant CAM-1 showed an increasing transepithelial resistance until about 6 h post infection, which later slighty reduced (Fig. 4a). Moreover, overexpression and complementation strains induced a prominent detrimental effect of the cells within 10 h of infection. The breakdown of the transepithelial resistance by CDC-E8392 pXMJ19_DIP0733 was considerably faster than by the complementation strain CAM-1 pXMJ19_DIP0733, which reached a lower basal resistance of around 200 Ω cm^− 2^ at just 6 h post infection (Fig. 4b). In comparison to the CAM-1 pXMJ19_DIP0733-cc strain, the transepithelial resistance of the infected cells with the CAM-1 pXMJ19_Cg0896 + cc was reduced dramatically (Fig. 4c). At 5 h post-infection, cells infected with the strain CAM-1 pXMJ19_Cg0896 + cc had already decreased the transepithelial resistance. On the other hand, cells infected with CAM-1 pXMJ19_DIP0733-cc remained with a high resistance of about 900–1000 Ω until 6 h and only after 11 h of infection was the transepithelial resistance of the cells reduced. The same tendency was observed for the CAM-1 pXMJ19_Cg0896 strain. These data further supported the important role of the DIP0733 coiled-coil region in host cell interaction and cell damage.

### *C. elegans* survival assay

**Fig. 5:**
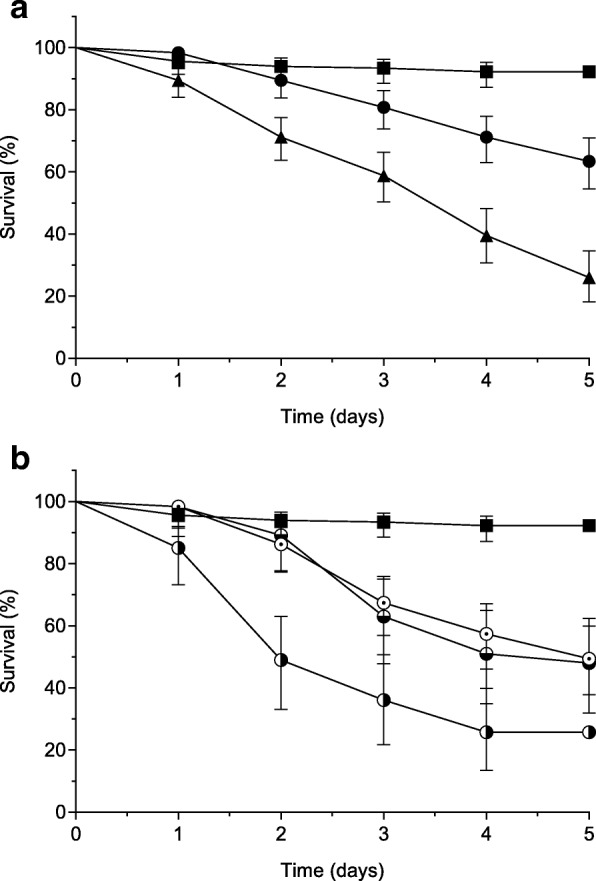
Nematode survival assay. Nematode survival was followed after infection with *E. coli* OP50 (■), *C. diphtheriae* CDC-E8392 (▲) and CAM-1 (●) (**a**), CAM-1 pXMJ19_Cg0896 (⊙), CAM-1 pXMJ19_DIP0733-cc (◒) and CAM-1 pXMJ19_Cg0896 + cc (◑) (**b**). Data are the mean of at least three parallel experiments with 20 worms per plate and error bars represent 95% CI

Previous study of *C. elegans* as a model for bacterial infection [14] revealed a high ability of nematode colonization and killing by the *C. diphtheriae* strain CDC-E8392 in comparison to the DIP0733 disruption mutant CAM-1, and this result was confirmed by the survival assay of this present study (*p* < 0.0001) (Fig. 5a). About 70% of the nematodes survived in contact with the mutant strain CAM-1, while around 70% were killed by the CAM-1 pXMJ19_Cg0896 + cc strain within 5 days (Fig. 5). Interestingly, the survival curve represented by the worms infected with the strain CAM-1 pXMJ19_Cg0896 + cc showed no significant difference compared to the strain CDC-E8392 (*p* = 0.576), while a significant difference was observed when compared to the CAM-1 (*p* < 0.0206). The mutant strain expressing *C. glutamicum* DIP0733 homolog (CAM-1 pXMJ19_Cg0896) and the truncated mutant CAM-1 pXMJ19_DIP0733-cc were less virulent to the nematodes, depicting similar survival rates between 50 and 60% within 5 days post-infection (Fig. 5). Moreover, no significant difference (*p* = 0.1651 for CAM-1 pXMJ19_Cg0896 and *p* = 0.0754 for CAM-1 pXMJ19_DIP0733-cc, respectively) was observed for both survival curves analyses when compared with CAM-1.

### Infection and monitoring of *G. mellonella*

**Fig. 6:**
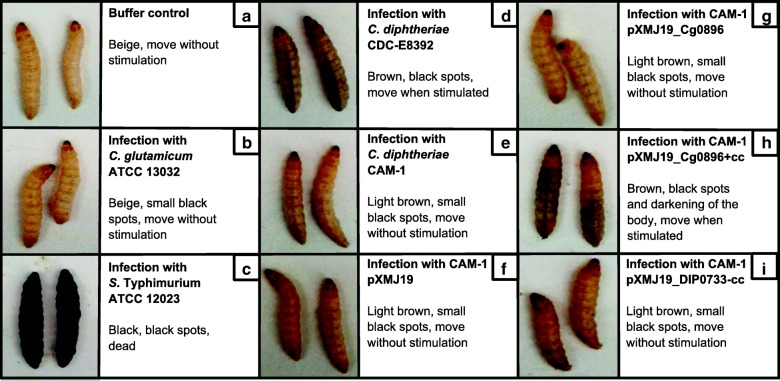
*G. mellonella* infection assay. *G. mellonella* larvae were injected with 10 μM MgSO_4_ buffer (**a**), non-pathogenic *C. glutamicum* ATCC 13032 (**b**), positive control for melanization *S.* Typhimurium ATCC12023 (**c**), *C. diphtheriae* strain CDC-E8392 (**d**), CAM-1 (**e**), CAM-1 pXMJ19 (**f**), and DIP0733 truncation mutants CAM-1 pXMJ19 Cg0896 (**g**), CAM-1 pXMJ19 Cg0896 + cc (**h**) and CAM-1 pXMJ19 DIP0733-cc (**i**)

**Fig. 7:**
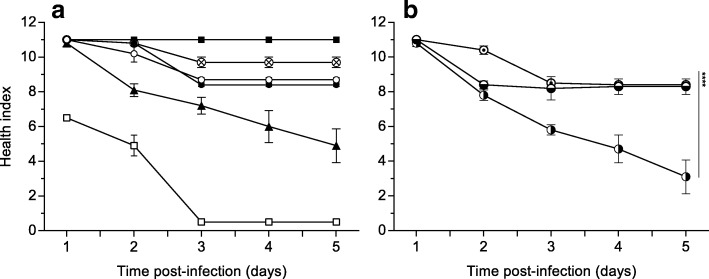
*G. mellonella* health index score monitoring. *G. mellonella* were monitored post-infection and the activity, melanization and survival features were scored according to their health index. The wax worms were inoculated with buffer (10 μM MgSO_4_), *S.* Typhimurium ATCC12023, non-pathogenic corynebacteria *C. glutamicum* ATCC 13032 (⊗), *C. diphtheriae* strain CDC-E8392 (▲), CAM-1 (●), CAM-1 pXMJ19 (o) (**a**), and the DIP0733 truncation mutants CAM-1 pXMJ19_Cg0896 (⊙), CAM-1 pXMJ19_Cg0896 + cc (◑) and CAM-1 pXMJ19_DIP0733-cc (◒) (**b**). Error bars represent mean ± SEM and 2-way ANOVA column statistics shows **** *p* < 0.0001

*G. mellonella* larvae are well established as a reliable insect model host to study the pathogenicity of corynebacteria [15]. As a qualitative approach, the melanization process after 3 days of infection is shown in Fig. 6. During the 5 days post-infection, the wax larvae were monitored daily and their activity, melanization and survival were quantitatively scored according to the health index scoring system (Additional file 2: Table S1; Fig. 7). While the control wax moth larvae (injected with buffer) remained the typical beige color and were highly active over all days, injection of the non-pathogenic *C. glutamicum* ATCC 13032 led to the development of small black spots after 3 days (Fig. 6a, b and Fig. 7a). After the second day, the melanization process of the larvae became more pronounced in response to injection of toxigenic *C. diphtheriae* CDC-E8392, which led to less active brown larvae with black spots in contrast to the injection of DIP0733 disrupted mutant strain CAM-1 (*p* < 0.0001) (Fig. 7a). Larvae infected with CAM-1 pXMJ19 Cg0896 were active and light brown in colour with the presence of small black spots in the body after the third day of infection (Fig. 6g and Fig. 7a). In contrast, larvae infected with the CAM-1 pXMJ19_Cg0896 + cc strain showed a lower and decreasing health index score due to the strong melanization with black spots, and a reduced activity over time (Fig. 6h and Fig. 7b) Larvae infected with the CAM-1 pXMJ19_DIP0733-cc strain led to small black spots in the posterior part of the lower abdomen one day earlier than when infected with the CAM-1 strain (*p* < 0.05) (Fig. 6i and Fig. 7). However, after 3 days of infection, their health index score remained very similar to those larvae infected with CAM-1, its control strain CAM-1 pXMJ19 and the strain CAM-1 pXMJ19_Cg0896 (Fig. 7). *S.* Typhimurium was previously validated for pathogenic studies in *G. mellonella* [31] and therefore it was used as a positive control of infection with high degree of melanization, immobility and rapid death also in this study (Fig. 6c and Fig. 7a).

## Discussion

The DIP0733 protein is a multi-functional virulence factor of *C. diphtheriae* that plays an important role in different interaction processes with host cells [13, 14]. Besides seven transmembrane helices, other annotated functional domains of DIP0733 have not been described until now. For a better understanding of the structure of DIP0733 and its function in the interaction with host cells, we investigated the SLiMs present among pathogenic and non-pathogenic homolog sequences. Our bioinformatic analysis showed that DIP0733 sequences from closely related pathogenic corynebacteria share higher similarities in SLiMs in contrast to non-pathogenic corynebacteria. While the role of SLiMs is well-characterized in eukaryotic intracellular signaling, in prokaryotic signaling it is less well-understood. Recently, the distribution of known and novel motifs in prokaryotic extracellular and virulence proteins across a range of bacterial species has been investigated [32]. Interestingly, many SLiMs present in virulence effector proteins were able to mimic eukaryotic motifs and facilitate the pathogen to control the intracellular processes of their hosts [32].

In this study, we investigated the role of the C-terminal coiled-coil domain of DIP0733 for pathogenicity of *C. diphtheriae*. Coiled-coil domains consist of two or more α-helical peptides that are enfolded around each other in a superhelical fashion [33]. They are ubiquitous folding motifs found in structural proteins and also play an important role in various intracellular regulation processes and membrane fusion [33]. In eukaryotes, this domain is present in transcription factors as well as fibrous proteins like keratin, myosin, epidermin and fibrinogen [33]. In prokaryotes, important coiled-coil domains are found as a part of murein lipoprotein in *E. coli*, colicin E3 in *S.* Typhimurium and Pep M5 protein in group A streptococci [33, 34].

DIP0733 plays a role as a MSCRAMM (microbial surface component recognizing adhesive matrix molecule) helping *C. diphtheriae* to colonize human tissues or to evade host immune mechanisms of bacterial clearance [14]*.* At a biochemical level, our results indicate that the coiled-coil domain of DIP0733 is important for the binding to Type I collagen and fibrinogen. Another *C. diphtheriae* protein, DIP2093 was recently described to be involved in binding to Type I, but not to Type IV collagen [12]. This indicates that DIP0733 is not acting alone as a MSCRAMM in *C. diphtheriae*.

Some bacteria, including non-toxigenic corynebacteria, have a natural tendency to adhere to biotic and/or abiotic surfaces and to form biofilms [7]. However, the mechanisms for bacterial adhesion orchestrated by DIP0733 and its coiled-coil domain differ concerning the binding to both biotic and abiotic material. Furthermore, our data corroborates with a previous study [14] and provides the evidence that the DIP0733 protein is involved in these processes but is not exclusively responsible for it.

Moreover, DIP0733 is also involved in adhesion to, invasion into epithelial cells and cell death [13, 14]. On the other hand, a recent study showed that a corresponding mutant for the DIP0733 homolog in *C. ulcerans* (CULC22_00609) showed no effect on adhesion to and invasion into epithelial cells [35]. Remarkably, this data corroborates with our study, as the coiled-coil domain of DIP0733 was predicted to be present only in *C. diphtheriae* and *C. pseudotuberculosis* and absent in *C. ulcerans.* Moreover, our data indicated that the C-terminal coiled-coil region of DIP0733 is crucial for adherence and internalization of *C. diphtheriae* to HeLa cells. Equivalent results were supported by detrimental effects by the break-down of transepithelial resistance of Detroit 562 cell monolayers after infection with *C. diphtheriae* wild-type and DIP0733 homologs as well as its truncated mutant strains. Whilst a dramatic break-down of transepithelial resistance was not observed for most of the *C. diphtheriae* strains tested by Ott and co-workers, *C. diphtheriae* DSM43989 showed the most severe effect after 3 h of infection [17], which was similarly demonstrated by the CDC-E8392 wild-type strain in this study. Conversely, other strain specific proteins and cellular niche factors may be involved in this process as similar effects were described for *C. ulcerans* 809 [35].

As an in vivo approach, the pathogenicity of DIP0733 and its C-terminal coiled-coil domain was further investigated and confirmed using *C. elegans* and *G. mellonella* as invertebrate animal model systems. Previous study showed that the DIP0733 mutant CAM-1 exhibited significant attenuation in nematode colonization, proliferation inside the worms and killing of the host compared to the wild-type CDC-E8392 [14]. Remarkably, CAM-1 expressing the *C. glutamicum* DIP0733 hybrid variant with C-terminal coiled-coil region was able to kill the nematodes, as similarly demonstrated by the complementation strain CAM-1 pXMJ19_DIP0733 [14]. A corresponding tendency concerning the influence of DIP0733 and its coiled-coil domain on bacterial pathogenicity in *G. mellonella* was observed in our study. Some entomopathogenic strains are able to inhibit the phenol-oxydase activation and therefore evade the melanization pathway [36]. However, most of the *G. mellonella* infection assays using a variety of Gram-positive bacteria, including *Streptococcus*, *Enterococcus*, *Staphylococcus* and *Listeria* spspecies (for review, see [37]) and importantly, pathogenic species of *Corynebacterium,* including *C. diphtheriae,* presented different degrees of melanization as an evidence for bacterial pathogenicity towards invertebrate model systems [15]. Remarkably, our data indicates a key role of DIP0733 and its coiled-coil domain in the melanization process induced by the infection with *C. diphtheriae*. Synthesis and deposition of melanin in *G. mellonella* function to encapsulate pathogens at the wound site followed by hemolymph coagulation and opsonization, analogous to abscess formation in mammalian infections [37, 38].

Our data showed the importance of the coiled-coil domain of DIP0733 in *C. diphtheriae* pathogenicity. Whether the coiled-coil domain is responsible for the interaction either with the host cells or with the holding of possible protein oligomers itself still remains unclear. To address this point, the protein crystallization and its oligomerization status must be further investigated.

## Conclusions

Taken together, DIP0733 is a multi-functional protein with an important C-terminal coiled-coil domain responsible for pathogenicity of *C. diphtheriae* in epithelial cells and invertebrate animal model systems.

## Additional files

Additional file 1: **Figure S1.** DIP0733 sequences alignment and identity scores. The detailed Multiple Sequences Alignment (MSA) of DIP0733 homologs of *C. diphtheriae* CDC-E8392, *C. diphtheriae* INCA 402, *C. ulcerans* BR-AD22, *C. pseudotuberculosis* 258 and *C. glutamicum* ATCC 13032 was anlyzed using Sequence Viewer 8.0 (a). For MSA, DIP0733 (Accession id: CAE49255) was used as query sequence and the compositional substitution matrix adjustment method was executed on BLASTp (b). (PDF 1059 kb)

Additional file 2: **Table S1.** Health index scoring system for *Galleria mellonella* adapted from Loh and co-workers (2013) [24]*.* Post-infection, *G. mellonella* larvae were monitored and scored daily for their activity, melanization and survival. Healthy, uninfected wax moth larvae typically score between 10 and 11, while infected dead wax moth larvae score between 0 and 1.5. (DOCX 14 kb)

## Abbreviations

CFU: Colony forming units
CLV: Cleavage sites
CSPD: Disodium 3-(4-methoxyspiro {1,2-dioxetane-3,2′-(5′-chloro)tricyclo [3.3.1.1^3,7^]decan}-4-yl)phenyl phosphate
DEG: Degradation sites
DMEM: Dulbecco’s modified Eagle’s medium
DOC: Docking sites
ECM: Extracellular matrix
ELM: Eukaryotic linear motif
FBS: Fetal bovine serum
HI: Heart infusion
LB: Luria Bertani
LIG: Ligand binding sites
MOD: Post-translational modification sites
MSCRAMM: Microbial surface component recognizing adhesive matrix molecule
NGM: Nematode growth medium
PBS: Phosphate buffered saline
SLiMs: Short linear motifs
TRG: Targeting sites
