# Supplementary material for: The C-terminal coiled-coil domain of Corynebacterium diphtheriae DIP0733 is crucial for interaction with epithelial cells and pathogenicity in invertebrate animal model systems
Source: BMC Microbiol. 2018 Sep 4;18:106. doi: 10.1186/s12866-018-1247-z (PMC6123952; doi:10.1186/s12866-018-1247-z)
Supplement: Supplementary file 1 — Figure S1. DIP0733 sequences alignment and identity scores. The detailed Multiple Sequences Alignment (MSA) of DIP0733 homologs of C. diphtheriae CDC-E8392, C. diphtheriae INCA 402, C. ulcerans BR-AD22, C. pseudotuberculosis 258 and C. glutamicum ATCC 13032 was anlyzed using Sequence Viewer 8.0 (a). For MSA, DIP0733 (Accession id: CAE49255) was used as query sequence and the compositional substitution matrix adjustment method was executed on BLASTp (b). (PDF 1059 kb) [file 12866_2018_1247_MOESM1_ESM.pdf]

a

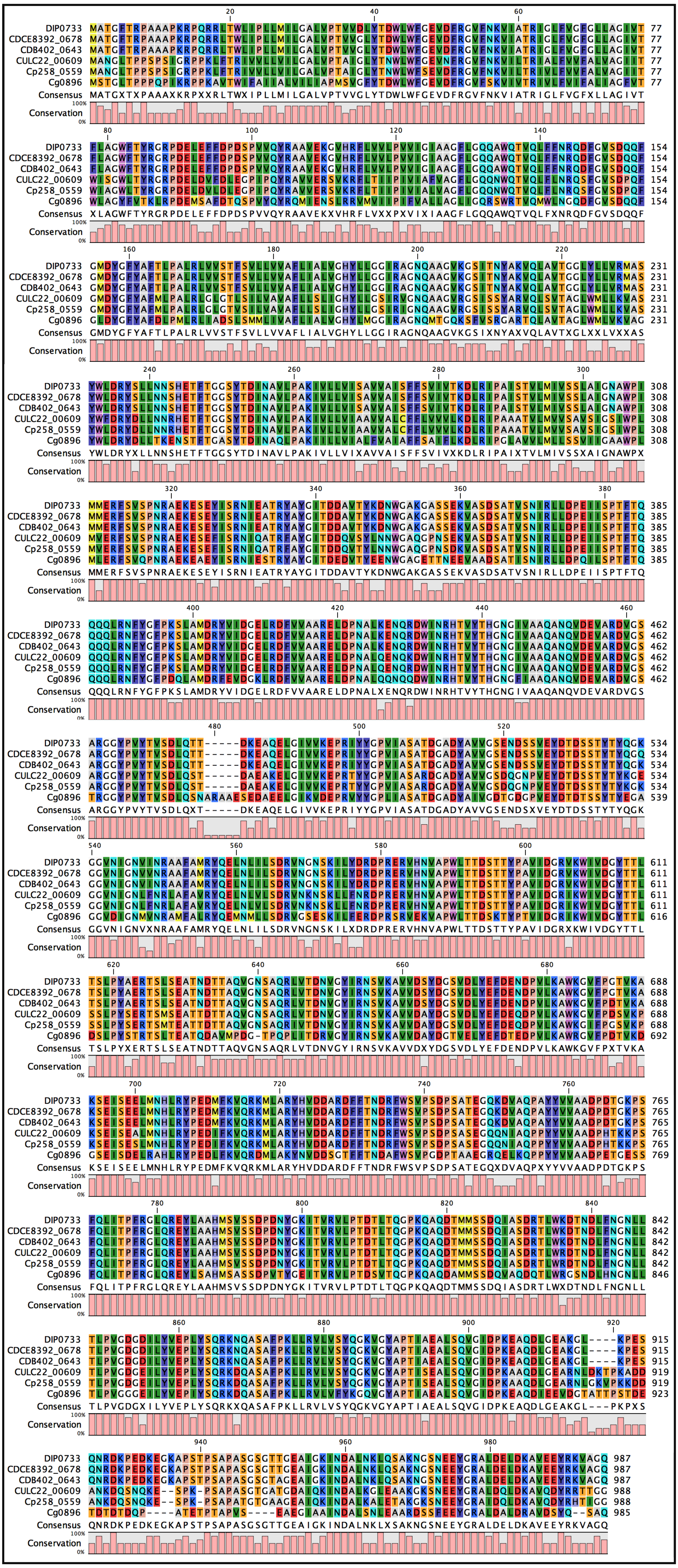

b

Strain	Accession	Query cover	Score	Identity	Gaps
C. diphtheriae CDC-E8392 *CDCE8392_0678*	AEX71676.1	100%	2010 bits (5207)	984/987(99%)	0/987(0%)
C. diphtheriae INCA 402 *CDB402_0643*	AEX45950.1	100%	2006 bits (5198)	984/987(99%)	0/987(0%)
C. ulcerans BR-AD22 *CULC22_00609*	AEG83325.1	99%	1666 bits (4315)	797/988(81%)	3/988(0%)
C. pseudotuberculosis 258 *Cp258_0559*	AFK16201.1	99%	1665 bits (4312)	798/990(81%)	7/990(0%)
C. glutamicum ATCC 13032 *Cg0896*	CAF19491.1	99%	1396 bits (3614)	670/987(68%)	10/987(1%)
