# Supplementary material for: The C-terminal coiled-coil domain of Corynebacterium diphtheriae DIP0733 is crucial for interaction with epithelial cells and pathogenicity in invertebrate animal model systems
Source: BMC Microbiol. 2018 Sep 4;18:106. doi: 10.1186/s12866-018-1247-z (PMC6123952; doi:10.1186/s12866-018-1247-z)
Supplement: Supplementary file 2 — Table S1. Health index scoring system for Galleria mellonella adapted from Loh and co-workers (2013) [24]. Post-infection, G. mellonella larvae were monitored and scored daily for their activity, melanization and survival. Healthy, uninfected wax moth larvae typically score between 10 and 11, while infected dead wax moth larvae score between 0 and 1.5. (DOCX 14 kb) [file 12866_2018_1247_MOESM2_ESM.docx]

| **Category** | **Description** | **Score** |
| --- | --- | --- |
| activity | no movement | 0 |
|  | minimal movement on stimulation | 1 |
|  | move when stimulated | 2 |
|  | move without stimulation | 3 |
| cocoon formation | no cocoon | 0 |
|  | partial cocoon | 0,5 |
|  | full cocoon | 1 |
| melanization | black larvae | 0 |
|  | black spots on black larvae | 0,5 |
|  | dark brown larvae | 1 |
|  | black spots on dark brown larvae | 1,5 |
|  | brown larvae | 2 |
|  | black spots on brown larvae | 2,5 |
|  | light brown larvae | 3 |
|  | small black spots on light brown larvae | 3,5 |
|  | ≥3 or spots on beige larvae | 4 |
|  | <3 or small spots on beige larvae | 5 |
|  | no melanization | 6 |
| survival | dead | 0 |
|  | alive | 2 |

**Additional file 2.**

**Table S1. Health index scoring system for *Galleria mellonella* adapted from Loh and co-workers (2013) [24]*.*** Post-infection, *G. mellonella* larvae were monitored daily for their activity, melanization and survival. A corresponding score was provided for each attribute that contributed toward an overall health index of an individual wax moth larvae. Healthy, uninfected wax moth larvae typically score between 10 and 11, while infected dead wax moth larvae score between 0 and 1.5.
